# A Novel Application of Buprenorphine Transdermal Patch to Relieve Pain in the Knee Joint of Knee Osteoarthritis Patients: A Retrospective Case-Control Study

**DOI:** 10.3390/jcm8071009

**Published:** 2019-07-10

**Authors:** Ho Young Gil, Sungchul Park, Na Eun Kim, Yi Hwa Choi, Jae Hyung Kim, Sooil Choi, Hyun Joong Kim, Jae Chul Koh, Byung Ho Lee, Sook Young Lee, Sang Kee Min, Bora Kim, Hye Seon Lee, Hae Won Jeong, Ji Hyun Park, Bumhee Park, Jong Bum Choi

**Affiliations:** 1 Department of Anesthesiology and Pain Medicine, Ajou University School of Medicine, Suwon 16499, Korea; 2 Department of Anesthesiology and Pain Medicine, Cha Bundang Medical Center, Cha University School of Medicine, Seongnam 13496, Korea; 3 Department of Anesthesiology and Pain Medicine, Inha University School of Medicine, Incheon 22332, Korea; 4 Department of Anesthesiology and Pain Medicine, Hallym Sacred Heart Hospital, Hallym University College of Medicine, Anyang 14068, Korea; 5 Department of Anesthesiology and Pain Medicine, Hallym Dongtan Sacred Heart Hospital, Hallym University College of Medicine, Hwaseong 18450, Korea; 6 Department of Anesthesiology and Pain Medicine, International St.Mary’s Hospital, Catholic Kwandong University, College of Medicine, Incheon 22711, Korea; 7 Department of Anesthesiology and Pain Medicine, Korea University Ansan Hospital, Ansan 15355, Korea; 8 Department of Anesthesiology and Pain Medicine, Korea University Anam Hospital, Seoul 02841, Korea; 9 Hwalgichan Orthopedic Surgery and Pain Clinic, Seoul 03329, Korea; 10 Office of Biostatistics, Ajou Research Institute for Innovative Medicine, Ajou University Medical Center, Suwon 16499, Korea; 11 Department of Biomedical Informatics, Ajou University School of Medicine, Suwon 16499, Korea

**Keywords:** buprenorphine, transdermal patch, osteoarthritis, knee, knee joint

## Abstract

Osteoarthritis (OA) is considered to be one of the most disabling diseases. The intra-articular opioid injection has been widely studied for its simplicity, safety, and efficacy in OA. In this study, however, we suggest a novel method of buprenorphine transdermal patch (BTDP) to painful knee joints of OA patients, instead of intra-articular opioid injection, and subsequently compared the knee application with conventional chest application. We retrospectively enrolled 213 patients with knee OA who did not respond to conventional therapy. The Numeric Rating Scale (NRS), adverse effects, and compliance were recorded before and after the application of the BTDP. All parameters were compared between the knee applied group and the chest applied group. After the BTDP application, the NRS score in the knee applied group was lower than that of the chest applied group (*p* = 0.007). NRS scores after buprenorphine patch decreased to 2.21 ± 0.77, and 2.55 ± 0.71 in the chest applied group and the knee applied group, respectively. The adverse effects were 19.32% in the knee applied group, and 64.00% in the chest applied group. The compliances were 82.95% and 37.60% in the knee applied group and chest applied group, respectively. This novel application of BTDP directly to the painful knee joint of knee OA patients led to a decrease in the NRS score, adverse effects, and an increase in compliance compared with the chest application method.

## 1. Introduction

Osteoarthritis (OA) is considered to be one of the most disabling diseases in developed countries [1], with pain being its most significant symptom, and it is often difficult to manage due to aggravation by weight-bearing and movement of the hip and knee joints. Medication including paracetamol, traditional nonsteroidal anti-inflammatory drugs (NSAIDs), and selective cyclooxygenase-2 inhibitors may be administered to relieve pain that originates from the joints; however, NSAIDs and selective cyclooxygenase-2 inhibitors also show many potentially serious side effects, especially in elderly patients [2]. The 2008 and 2009 guidelines recommend the use of weak opioids or low doses of strong opioids when paracetamol, with or without topical NSAIDs, does not sufficiently relieve pain [2,3]. However, although several randomized controlled studies have shown analgesic effects in musculoskeletal diseases, adverse effects are also commonly observed, and about 50% of patients discontinue opioid treatment due to these adverse effects [4,5].

**Figure 1:**
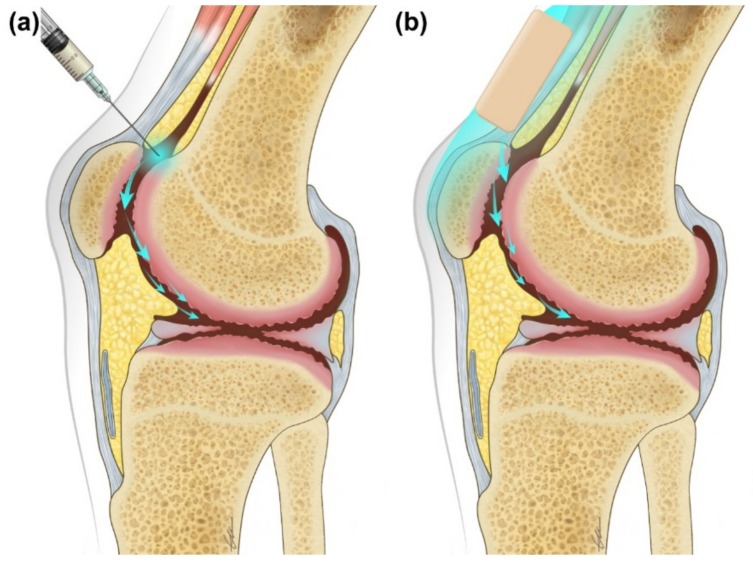
(**a**) Intra-articular injection of opioids in the painful knee joint of knee osteoarthritis patients, (**b**) application of the buprenorphine transdermal patch on the painful knee joint of knee osteoarthritis patients (Jong’s hypothesis).

Concurrently, the intra-articular (IA) injection of opioids has been studied for some time (Figure 1). Opioid binding receptors have been identified within the synovium of joint spaces, suggesting that an analgesic effect from the opioid injected into the IA space may be locally mediated [6]. When injected at the end of arthroscopic surgery, the IA opioid could reduce postoperative pain through peripheral opioid receptors [7]. Furthermore, another opinion suggested that inflammation would destroy the perineurium of the nerve, and opioids would be better transported to opioid receptors due to the destroyed perineurium. It has also been reported to reduce pain through these pathways [8], such as inflammatory reaction [9,10,11].

**Figure 2:**
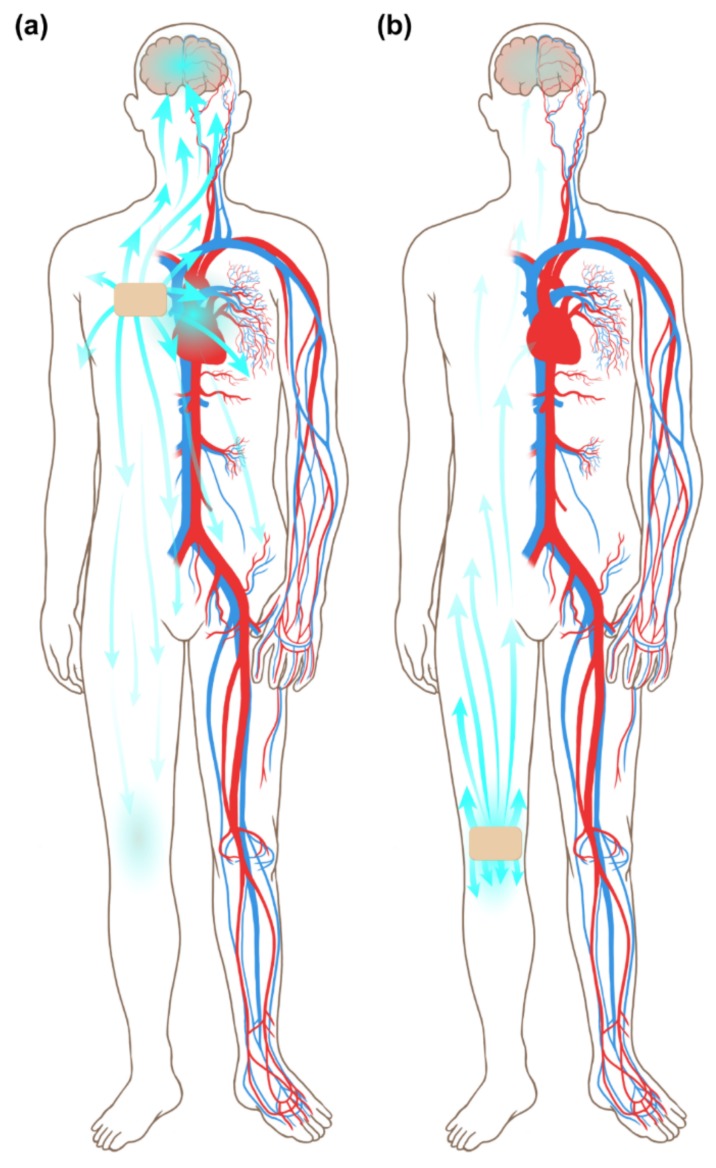
(**a**) Application of the buprenorphine transdermal patch on the chest of knee osteoarthritis patients (conventional application method), (**b**) application of the buprenorphine transdermal patch on the painful knee joint of knee osteoarthritis patients.

Here, we suggest a novel method of application of a buprenorphine transdermal patch instead of the IA opioid injection, with the hypothesis that the application of this opioid transdermal patch on painful joints would transport the specific opioid component to joint spaces by diffusion (Figure 1). This hypothesis has subsequently been named Jong’s hypothesis after Jong Bum Choi, the originator. In this study, we applied the buprenorphine transdermal patch to the painful knee joint of knee OA patients. The purpose of this research is to study the analgesic effects, adverse effects, and compliance of the buprenorphine transdermal patch applied to painful knee joints of knee OA patients, and to compare the knee applied group with the conventional chest applied group with respect to all parameters (Figure 2).

## 2. Materials and Methods

This study was a retrospective case-control study. Reviews of medical records were approved by the Institutional Review Board of the Ajou University Hospital of Korea (IRB No. AJIRB-MED-MDB-19-069), and registered at ClinicalTrials.gov (Identifier: NCT03947125) in May 2019. The requirement for informed consent was waived because of the retrospective case-control nature of the study.

### 2.1. Setting

This research was performed in Ajou University School of Medicine in Suwon, Republic of Korea. The medical records were retrieved from January 2018 to December 2018. Recruitment and data collections were performed in April, 2019.

### 2.2. Participants

**Figure 3:**
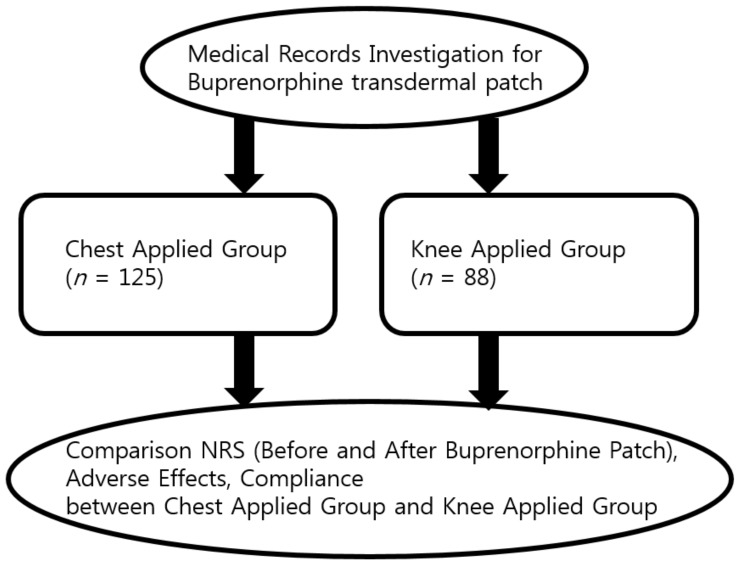
A flow diagram of research. NRS, Numeric Rating Scale.

Through medical records, we retrospectively enrolled 213 patients with OA of the knee who did not respond to conventional therapy. The inclusion criteria were as follows: (1) Age over 19 years; (2) diagnosis of OA of the knee by radiographic evidence of the aforementioned OA, as defined by Grades II to IV of the Kellgren and Lawrence scale [12]; (3) pain score over 4 in the Numeric Rating Scale (NRS) from the relevant joint; and (4) buprenorphine transdermal patch prescribed for unilateral OA of the knee. The exclusion criteria were as follows: (1) History of total knee replacement surgery; (2) treatment with weak or strong opioid analgesics; (3) contraindications to treatments with opioid medication, such as a history of alcohol or substance abuse; (4) clinically significant systemic disease or any reduced organ function; (5) use of antidepressants, antiepileptic drugs, steroids, and/or hypnotics (that may have increased respiratory depression of buprenorphine) and; (6) missing data. Patients were divided into two groups, with the control group consisting of patients with a chest applied buprenorphine transdermal patch, and the other group consisting of patients with a painful knee joint applied with a knee applied buprenorphine transdermal (Figure 3). Buprenorphine transdermal patchs were replaced every seven days.

### 2.3. Clinical Evaluations

The NRS was checked before and at one month after the prescription of the buprenorphine transdermal patch. NRS before the buprenorphine patch was the average score across 1 week before the administration of the BTDP buprenorphine starts. NRS after buprenorphine patch was the average score during the administration of the buprenorphine patch. After buprenorphine patch was detached, NRS was not evaluated. The adverse effects of the buprenorphine transdermal patches were checked and recorded, while the compliance of the buprenorphine transdermal patch was also investigated. All parameters were then compared between the chest applied group and knee applied group.

### 2.4. Statistical Analysis

The sample size was 205 from a pilot study with a 15% drop out rate, which achieved 80.049% power, and a mean difference of −0.3, with a standard deviation of 0.3 for both groups, and with a significance level (alpha) of 0.050. The Student’s t-test was used to compare the chest applied group and knee applied group in terms of the demographic data, while the Mann-Whitney U test, Chi-squared test, and the Fisher’s exact tests were used to compare the two groups in terms of the NRS, adverse effects, compliance, and dose of buprenorphine administered. All data was analyzed both in intention to treat, and in per protocol. All statistical analyzes were performed using R software, version 3.5.1. A *p*-value less than 0.05 was considered to be statistically significant.

## 3. Results

**Figure 4:**
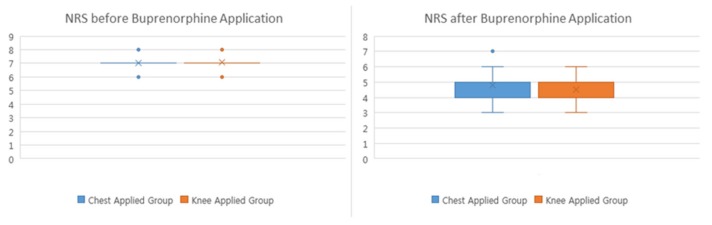
Numeric rating scale (NRS) before buprenorphine transdermal patch application.

The demographic data are shown in Table 1. In intention to treat analysis, the NRS in the knee applied group after the buprenorphine transdermal patch application was lower than that of the chest applied group (Table 2, Figure 4, *p* = 0.007). In per protocol analysis (except patients stopping buprenorphine transdermal patch), there were no differences between the chest applied group and the knee applied group (Table 3). Furthermore, in the knee applied group, the total percentage of adverse effects was 19.32% (17/88, gastrointestinal (GI): 2, genitourinary: 1, and dermatological: 14) and in the chest applied group, it was 64.00% (80/125, gastrointestinal: 33, central nervous system (CNS): 24, skin problem: 15, and others: 8) (Table 4, *p* < 0.001). The compliance rates were 82.95% (73/88) and 37.60% (47/125) in the knee applied group and the chest applied group, respectively (Table 2, *p* < 0.001). The reasons for discontinuation were due to adverse effects, but a few patients with adverse effects continued to keep buprenorphine patch because of pain. The doses of buprenorphine of patient maintaining the patch were 6.71 ± 2.91 μg/h, and 7.66 ± 4.40 μg/h in the knee applied group and the chest applied group, respectively (Table 5, *p* = 0.387), while the doses of buprenorphine in patients stopped were 7.00 ± 4.14 μg/h, and 6.99 ± 3.72 μg/h during the period when the BTDPs were applied in the knee applied group, and the chest applied group, respectively (Table 5, *p* = 0.869). Effect size for the Mann-Whitney U test by R was 0.37 in comparison of NRS after buprenorphine patch.

## 4. Discussion

In this study, we found that our method of application of the buprenorphine transdermal patch directly to the painful knee joint of knee OA patients is more effective in reducing pain, and also less likely to have adverse effects compared with the conventional chest application method. Furthermore, the compliance was significantly higher in the knee applied group. These results allow for us to speculate that the absorption of buprenorphine might be greater at the local opioid receptors present in knee joints than when entering the systemic circulation.

In a previous study of 114 patients with chronic nonmalignant musculoskeletal pain, 50 patients (43.90%) stopped applying the buprenorphine transdermal patches due to the adverse effects observed, and subsequently dropped out. These adverse effects (78.10%) were generally mild or moderate, and mostly included nausea (39.50%) and constipation (31.60%), followed by dizziness (27.20%), somnolence (19.30%), vomiting (16.70%), headache (8.80%), pruritus (7.90%), and application site reactions (6.10%) [13]. Similar results were observed in our study. In the chest applied group, adverse GI and CNS effects were observed in 33 cases (41.25%) and 24 cases (30.00%), respectively; in contrast, there were no CNS cases, and only two GI cases (11.76%) of adverse effects that had occurred in the knee applied group.

According to a review of previous studies of IA morphine injection after knee arthroscopy, the analgesic effect of morphine is mediated by local opioid receptors in the joint [7]; here, its poor lipid solubility would hamper its passage across the synovial membrane into the bloodstream [14]. The plasma morphine levels are much lower than what is generally accepted to be necessary for a systemic analgesic effect [15,16,17]. Additionally, the partial agonist effect of buprenorphine at the µ-opioid receptor further reduces the adverse CNS and GI effects, along with having a ceiling effect for respiratory depression but not for analgesia, which results in a reduced risk for this potentially fatal adverse event as compared to other full opioid agonists [18,19]. With respect to the adverse skin effects, the ratio of adverse skin effects to the total adverse effects was higher in the knee applied group, but the incidence was not much higher compared with the control group. This is due to the fact that when the patches are applied directly to a painful knee, the surface area of the skin that it can be attached to is limited. Nevertheless, the incidence of such adverse skin reactions was not significantly higher compared with the conventional method; therefore, patients may be recommended our new application method without major issues.

Two other pieces of research showed an effect size of 0.37 [20,21]. The effect size of our results were 0.37, the same as the cut-off values shown in other research, therefore, a decision regarding the clinical relevance of the NRS scores could not be made.

Our findings need to be interpreted within the limitations of the study: (1) We could not analyze the concentration of buprenorphine directly by aspirating the joint fluid. Comparing the differences in concentrations between both groups would further support our hypothesis. (2) We could not analyze the differences in adverse effects due to dose differences. (3) This study is retrospective in nature, and has a relatively small sample size. For decreasing large placebo effects and verifying treatment effects, prospective randomized clinical trials with identically appearing placebo patches would be required in the future. (4) The effect size of this study were the same as the cut-off values from previous research, so a decision regarding its clinical relevance could not be made. This would need to be investigated in a further study. (5) This study did not exclude the effect of some medications (NSAIDs, acetaminophen and muscle relaxant, etc.) in participants.

## 5. Conclusions

In knee OA patients, the regional application of the buprenorphine transdermal patch directly to the painful knee joint showed an increase in effectiveness, compliance, and a decrease in adverse effects compared with the conventional chest application method.

## Figures and Tables

**Table 1:** Demographic data.

**Variable**	**Chest Applied Group** **(*n* = 125)**	**Knee Applied Group** **(*n* = 88)**	***p*-Value**
Sex			1.000 ^1^
Male	63 (50.40)	44 (50.00)	
Female	62 (49.60)	44 (50.00)	
Age, years	62.25 ± 15.44	57.00 ± 15.78	0.018 ^2,^*
Height, cm	161.89 ± 9.46	164.15 ± 10.45	0.180 ^3^
Weight, kg	63.04 ± 13.18	64.82 ± 12.12	0.465 ^2^
BMI, kg/m^2^	23.86 ± 4.12	24.00 ± 3.39	0.826 ^3^
KL grade ^4^	II:59, III:61, IV:5	II:44, III:39, IV:5	0.892
Malalignment (Varus, Valgus)	95 (76.00)	69 (78.41)	0.681

Data are presented as *n* (%), Mean ± Standard Deviation for the numerical variables. ^1^ Chi-squared test was used. ^2^ Mann-Whitney test was used. ^3^ Student’s t-test was used. * *p* value < 0.05. BMI: Body mass index. KL grade ^4^, Kellgren Lawrence grade.

**Table 2:** Comparison of the Numeric Rating Scale (NRS), adverse effects, and compliance between the chest applied group and knee applied group. (Intention to treat analysis).

Variable	Chest Applied Group (*n* = 125)	Knee Applied Group (*n* = 88)	*p*-Value
NRS before			0.195
buprenorphine			
application			
Mean ± SD	7.00 ± 0.31	7.06 ± 0.32	
Median (Q1, Q3)	7 (7.00, 7.00)	7 (7.00, 7.00)	
NRS after			0.007 *
buprenorphine			
application			
Mean ± SD	4.79 ± 0.81	4.51 ± 0.69	
Median (Q1, Q3)	5 (4.00, 5.00)	4 (4.00, 5.00)	
Side effect			<0.001 *
No	45 (36.00)	71 (80.68)	
Yes	80 (64.00)	17 (19.32)	
Maintenance			<0.001 *
No	78 (62.40)	15 (17.05)	
Yes	47 (37.60)	73 (82.95)	

Data are presented as *n* (%), Mean ± Standard Deviation for the numerical variables. Mann-Whitney U test was used for the numerical variables and Chi-squared test was used for the categorical variables. * *p* value < 0.05. NRS, Numeric Rating Scale.

**Table 3:** Comparison of the NRS, adverse effects, and compliance between the chest applied group and knee applied group (per protocol analysis).

Variable	Chest Applied Group (*n* = 47)	Knee Applied Group (*n* = 73)	*p*-Value
NRS before buprenorphine application			0.470
	7.09 ± 0.35	7.04 ± 0.31	
	7 (7.00, 7.00)	7 (7.00, 7.00)	
NRS after buprenorphine application			0.584
	4.53 ± 0.75	4.45 ± 0.69	
	4 (4.00, 5.00)	4 (4.00, 5.00)	
Side effect			0.644
No	45 (95.74%)	71 (97.26%)	
Yes	2 (4.26%)	2 (2.74%)	

Data are presented as *n* (%), Mean ± Standard Deviation and Median (Q1, Q3) for the numerical variables. Mann-Whitney U test was used for the numerical variables and Fisher’s exact test was used for the categorical variables. * *p* value < 0.05. NRS, Numeric Rating Scale.

**Table 4:** Comparison of the adverse effects between the chest applied group and knee applied group.

Variables	Chest Applied Group	Knee Applied Group	*p*-Value
Gastrointestinal system	33 (41.25)	2 (11.76)	
nausea	26	1	
vomiting	1	0	
constipation	4	0	
dry mouth	2	1	
Central nervous system	24 (30.00)	0 (0.00)	
dizziness	18	0	
somnolence	6	0	
Cardiovascular system	0 (0.00)	0 (0.00)	
Genitourinary system urinary retention	0 (0.00) 0	1 (5.88) 1	
Respiratory system	0 (0.00)	0 (0.00)	
Skin and appendages dermatitis irritation	15 (18.75) 6 9	14 (82.35) 4 10	
Special senses	0 (0.00)	0 (0.00)	
Body as a whole	0 (0.00)	0 (0.00)	
Metabolic and nutritional system	0 (0.00)	0 (0.00)	
Others general weakness	8 (10.00) 8	0 (0.00) 0	
Total	80/125(64.00)	17/88(19.32)	<0.001 *

Data are presented as *n* (%). Fisher’s exact test was used. * *p* value > 0.05.

**Table 5:** Comparison of the buprenorphine doses administered between the chest applied group and knee applied group.

Variable	Chest Applied Group	Knee Applied Group	*p*-Value
Buprenorphine dose of stopping group			0.869
*n*	78	15	
Mean ± SD	6.99 ± 3.72	7.00 ± 4.14	
Buprenorphine dose of maintenance group			0.387
*n*	47	73	
Mean ± SD	7.66 ± 4.40	6.71 ± 2.91	

Data are presented as Mean ± Standard Deviation. Mann-Whitney U test was used. SD, Standard Deviation.
